# Tendon transfers in the setting of shoulder arthroplasty

**DOI:** 10.1016/j.xrrt.2024.03.007

**Published:** 2024-04-07

**Authors:** Joseph G. Monir, Eric R. Wagner

**Affiliations:** a Orlando Health Jewett Orthopedic Institute, Orlando, FL, USA; b Emory University School of Medicine, Atlanta, GA, USA

**Keywords:** Shoulder, Total shoulder arthroplasty, Tendon transfer, Reverse, Anatomic, Latissimus transfer, Allograft prosthetic composite, Revision shoulder arthroplasty

## Abstract

**Background:**

Tendon transfers in conjunction with reverse total shoulder arthroplasty can significantly improve functional outcomes in patients with glenohumeral arthritis and irreparable rotator cuff deficiency. There have been multiple promising new techniques described within the last 20 years that shoulder surgeons should become familiar with.

**Methods:**

The authors reviewed the literature on tendon transfers in the setting of reverse total shoulder arthroplasty. Procedures to restore various shoulder functions were described including surgical anatomy, techniques, pearls and pitfalls, and photos.

**Results:**

Subscapularis insufficiency can be reconstructed with a pectoralis major transfer or latissimus dorsi transfer, with the latter having better clinical outcomes and a more anatomic line of pull. Posterosuperior rotator cuff deficiency can be reconstructed with a latissimus transfer (L’Episcopo transfer) or lower trapezius transfer, with the latter proving superior in biomechanical and short-term studies. Deltoid deficiency can be reconstructed with a pedicled upper pectoralis major transfer. Massive proximal humerus bone loss can be reconstructed with an allograft-prosthetic composite, and any of the aforementioned transfers can be utilized in this setting as well.

**Conclusion:**

Tendon transfers in conjunction with reverse shoulder arthroplasty can significantly improve functional outcomes in patients with glenohumeral arthritis and irreparable rotator cuff deficiency. There have been multiple promising new techniques described within the last 20 years that shoulder surgeons should become familiar with.

Total shoulder arthroplasty involves prosthetic replacement of both the humeral head and glenoid. Anatomic total shoulder arthroplasty (aTSA) was first performed by Péan^36^ in 1893 to treat a patient with tuberculosis, and it was subsequently popularized by Neer^34^ in 1963. As the indications and popularity of aTSA grew, it became evident that these prostheses required a competent rotator cuff for optimal function.^1^^,^^52^

The reverse total shoulder arthroplasty (rTSA) was designed by Grammont^21^ as a potential treatment for patients with incompetent rotator cuffs. The Grammont rTSA medialized the center of rotation. This gave the deltoid an improved moment arm, allowing it to compensate for the function of the incompetent rotator cuff. Additionally, by inverting the anatomic ball-and-socket design and having a semiconstrained glenohumeral articulation, the rTSA had greater inherent stability than the aTSA in the absence of the dynamic stability normally provided by the rotator cuff.

The modifications and improvements to Grammont’s original design have led to exponential increases in the number of total shoulder arthroplasties done over the past few years.^17^^,^^48^ While rTSA has been largely successful in improving function in patients with irreparable rotator cuff tears,^37^ the continued desire to further restore lost shoulder function has recently led to increased interest in the use of tendon transfers about the shoulder. These tendon transfers can be performed in conjunction with shoulder arthroplasty, with anatomic or reverse total shoulder arthroplasties. A variety of transfers exist to augment the deficient shoulder girdle musculature in both aTSA and rTSA. These tendon transfers can allow patients who otherwise may not be ideal candidates for shoulder arthroplasty to not only obtain reasonable pain relief but also maximize potential functional deficits.

As the use of shoulder arthroplasty continues to expand at exponential rates,^17^^,^^48^ familiarity with these tendon transfers will allow shoulder surgeons to better care for increasingly complex patients. In this review, the authors will discuss the indications, techniques, and outcomes of various tendon transfers about the shoulder girdle that can be utilized in combination with shoulder arthroplasty.

## Subscapularis insufficiency

aTSA relies on a functioning rotator cuff to reproduce native shoulder biomechanics. Subscapularis insufficiency has traditionally been viewed as a contraindication for aTSA due to the altered biomechanics leading to superior migration and a higher risk for anterior escape.^45^ Patients with glenohumeral arthritis in the setting of subscapularis insufficiency are commonly directed to rTSA due to its ability to compensate for a deficient subscapularis. However, functional outcomes of aTSA can be superior to those of rTSA, even with similar rotator cuff status.^33^ Tendon transfers to restore subscapularis function have therefore been proposed to allow patients who are otherwise well-suited to undergo aTSA to still receive the procedure. Furthermore, many young patients who underwent a hemiarthroplasty due to their young age might not be the ideal candidate for a revision to rTSA. In these patients, revising the hemiarthroplasty to an aTSA with a tendon transfer to either augment the already repaired subscapularis or replace a torn subscapularis has the benefits of potentially improved functional outcomes and future options if another revision surgery is required in the future.

### Pectoralis major transfer

Pectoralis major transfer (PMT) has historically been the preferred tendon transfer to restore subscapularis function. It was first described by Wirth and Rockwood^53^ in 1997 to treat irreparable subscapularis tears, and it has since been adapted for use in conjunction with shoulder arthroplasty. Multiple surgical technique variations exist, but the most commonly utilized is that described by Resch et al,^40^ where the superior one-half to two-thirds of the pectoralis major tendon are passed posterior to the conjoint tendon and inserted onto the lesser tuberosity.

In their systematic review in 2016, Shin et al^43^ reviewed pooled functional outcomes where possible. They found that PMT was associated with statistically significant improvements in shoulder abduction and forward flexion (FF), a reduction in postoperative external rotation (ER), and no change in internal rotation (IR) range of motion. Despite promising functional outcomes, there are concerns regarding PMT failure. Ortmaier et al^35^ described a series of patients with failed PMT, all successfully treated with rTSA. Elhassan et al^11^ specifically examined the outcomes of PMT after shoulder arthroplasty and found insignificant improvements in outcomes and a high failure rate.

### Latissimus dorsi transfer

**Figure 1:**
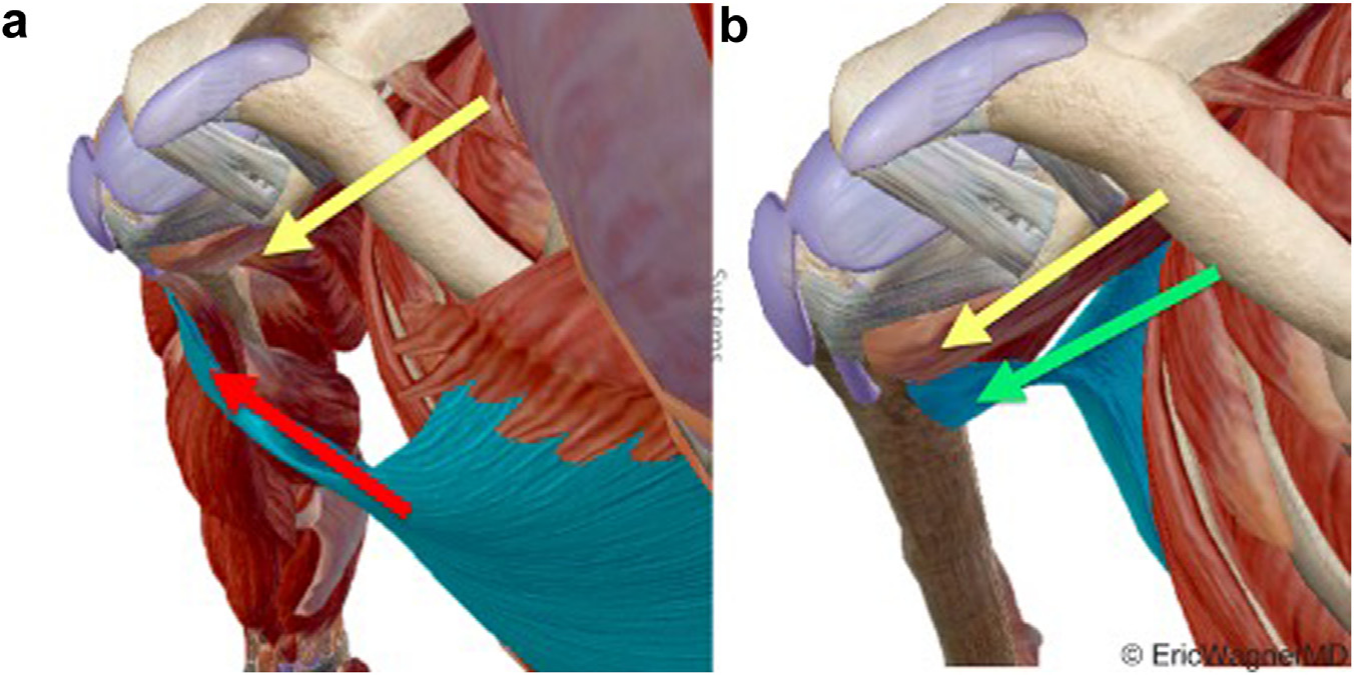
(**a**) Comparison of lines of pull of pectoralis major () and (**b**) latissimus dorsi () with subscapularis (). The latissimus dorsi transfer’s line of pull is closest to the native subscapularis.

Latissimus dorsi transfer (LDT) was initially described in 2014 as a more anatomical alternative to PMT.^10^ The latissimus dorsi originates on the posterior chest wall and has a line of pull nearly identical to the subscapularis. In contrast, the pectoralis major originates on the anterior chest wall, resulting in a line of pull that is nearly perpendicular to the native subscapularis (Fig. 1).

**Figure 2:**
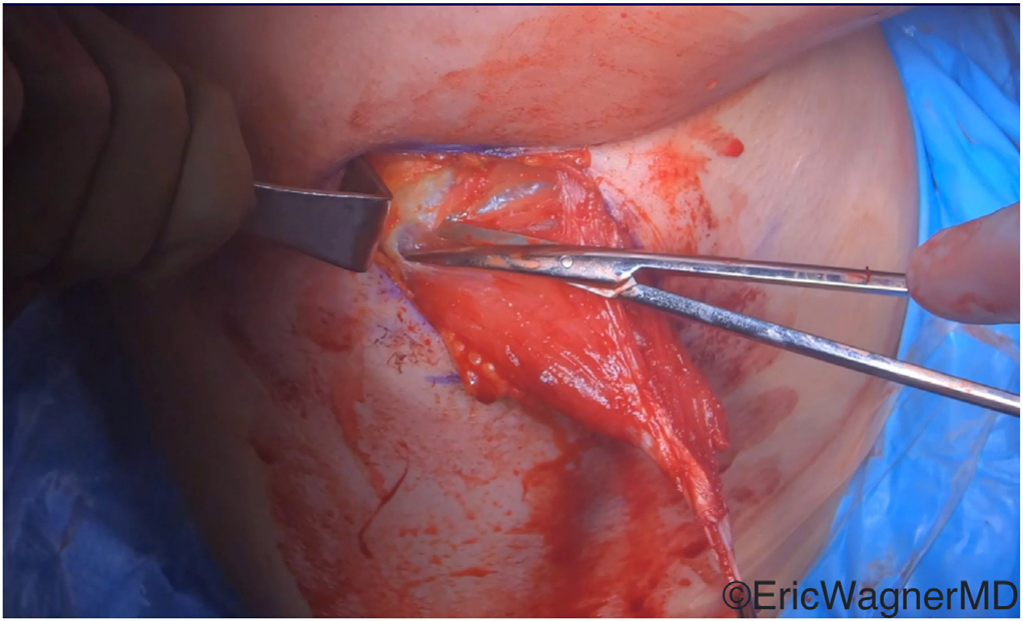
The latissimus dorsi tendon is released off its humeral insertion, and the muscle belly is mobilized by the lysis of adhesions.

**Figure 3:**
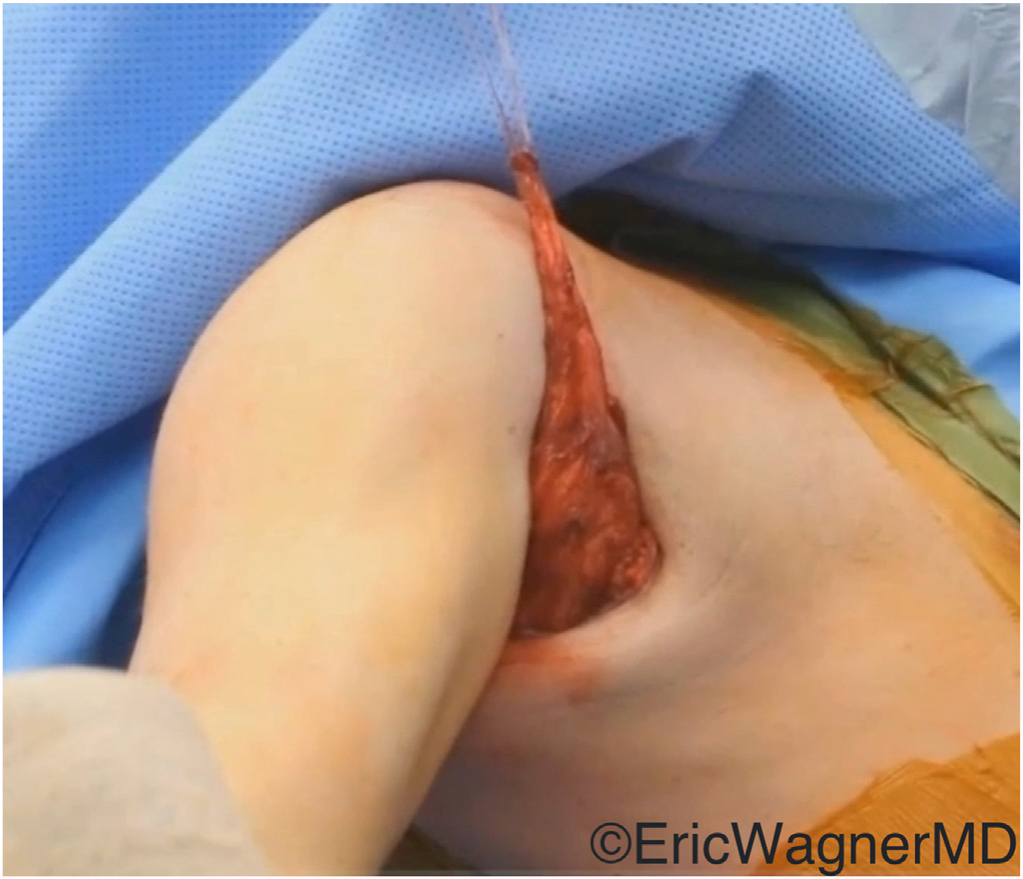
This figure demonstrates that the latissimus dorsi has been appropriately mobilized for anterior arthroscopic transfer to the lesser tuberosity.

**Figure 4:**
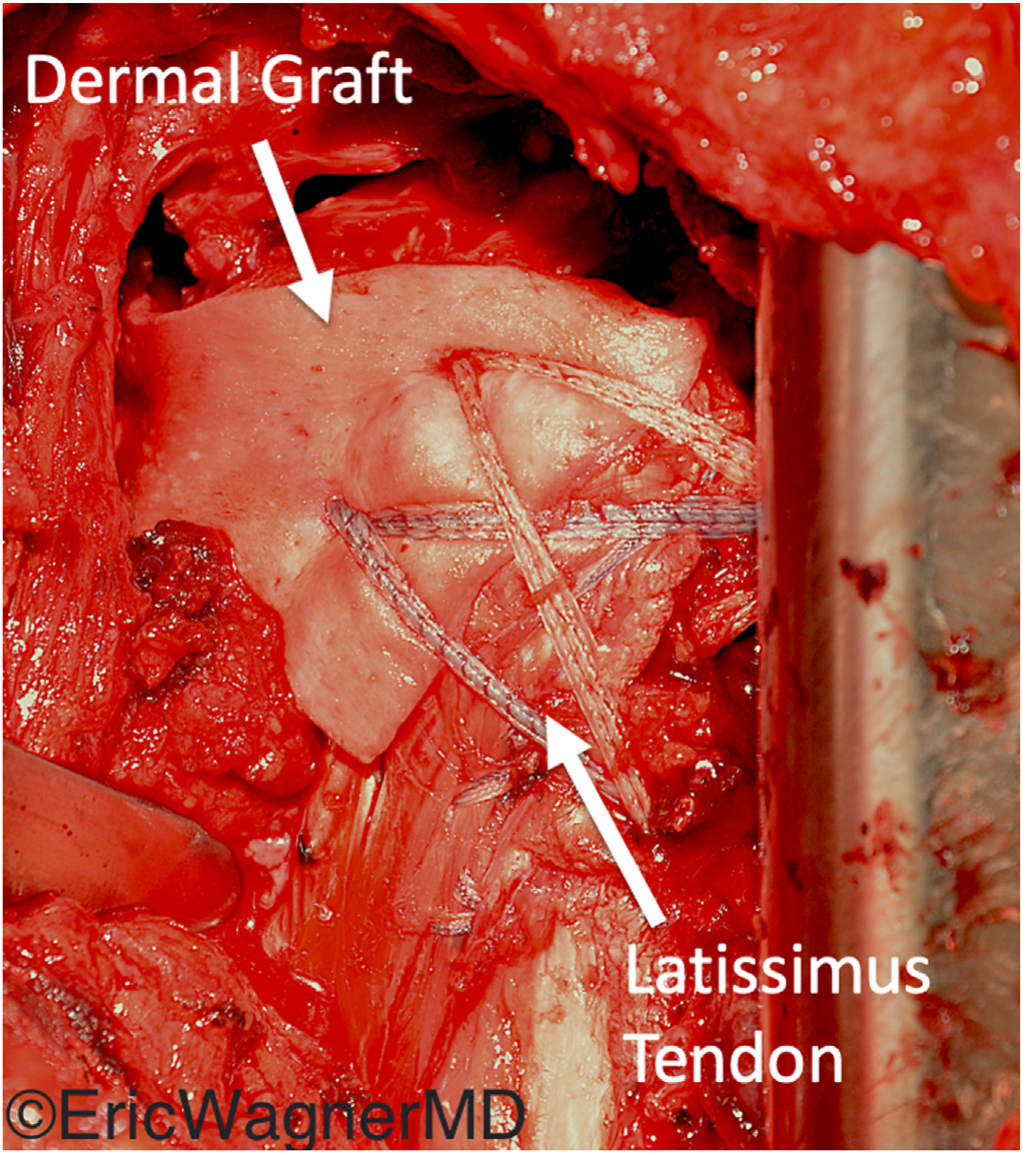
Latissimus dorsi transferred to the lesser tuberosity for subscapularis deficiency. The transfer was augmented in this case with a dermal allograft anterior capsular reconstruction sutured to the remnant subscapularis.

The surgical technique involves mobilizing the tendon of the latissimus dorsi from its insertion on the medial aspect of the bicipital groove (Fig. 2) and transferring it to either the superior or superolateral aspects of the lesser tuberosity. This procedure can be done via an open deltopectoral approach anteriorly in the setting of an arthroplasty or via an arthroscopic-assisted manner in the native shoulder^10^^,^^14^ (Fig. 3). Care must be taken to protect the radial nerve as it passes anterior to the latissimus dorsi. It will invariably lie within the surgical field and can be injured either directly or by overzealous retraction. In cases of arthroplasties, we also prefer to augment this transfer with an anterior capsular reconstruction from the anterior glenoid to the lesser tuberosity to assist with the initial resistance to anterior translation of the humeral head^8^ (Fig. 4).

Mun et al^32^ described a series of 24 patients who underwent open LDT and found significant improvement in functional outcomes and patient-reported outcomes measures (PROMs) with no complications or retears during the study period. Elhassan,^3^ Kany et al^24^ and Reinares et al^39^ demonstrated the feasibility of performing this LDT arthroscopically-assisted. To our knowledge, no one has investigated the outcomes of performing this LDT concomitantly with shoulder arthroplasty.

## Posterosuperior rotator cuff insufficiency

The posterosuperior rotator cuff, involving the supraspinatus and infraspinatus, provides the critical superior and posterior aspects of the force couple that maintains dynamic glenohumeral joint stability. Insufficient posterosuperior rotator cuff leads to superior humeral head migration from a loss of force couple balance as well as a loss of shoulder ER.

### Latissimus dorsi transfer (L’Episcopo)

L’Episcopo originally described transferring the latissimus dorsi and teres major tendons to restore shoulder ER for obstetric brachial plexus injuries in 1934.^26^ This transfer has subsequently been modified and applied to other clinical scenarios, and today it offers a powerful treatment for posterosuperior rotator cuff insufficiency.

**Figure 5:**
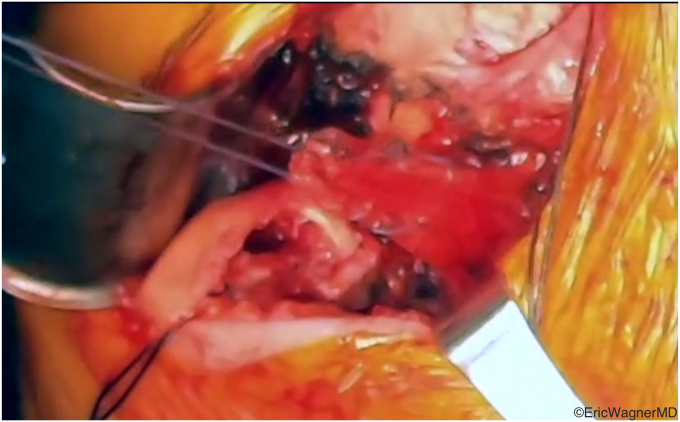
The latissimus dorsi tendon has been harvested and tagged through a standard deltopectoral approach. This is done prior to placement of the humeral stem.

Importantly, this transfer is different than the LDT discussed above. LDT for subscapularis insufficiency involves the transfer of the latissimus dorsi tendon to the lateral lesser tuberosity anterior to the humerus, allowing it to act as both an internal rotator and an anterior stabilizer. In contrast, the L’Episcopo transfer involves the transfer of the latissimus dorsi posterior to the humerus and insertion onto the lateral aspect of the proximal humerus near the teres minor and inferior infraspinatus insertions (Figs. 5 and 7). A modification to this transfer by Gerber is to anchor the latissimus tendon on the greater tuberosity near the superior infraspinatus insertion.^18^^,^^20^ These both modify its line of pull and turn the latissimus dorsi into an external rotator. The L’Episcopo allows for a more natural ER moment, while the modification by Gerber provides more superior and posterior stability in rebalancing the posterior force couple of the shoulder.

Multiple authors have described outcomes of the L’episcopo with or without concomitant teres major transfer in the setting of rTSA.^2^^,^^3^^,^^43^ These studies showed significantly improved active FF, active ER, visual analog scale scores, and improvements on multiple PROMs. Many studies have also shown very promising outcomes with the Gerber modification,^19^^,^^20^ and a recent systematic review showed very reliable outcomes with both techniques.^4^ Subscapularis insufficiency is a relative contraindication to the L’Episcopo transfer, as the transfer may compromise IR function in these patients. Furthermore, even when performed with rTSA, this can lead to a potential increased risk of anterior instability by taking away one of the few remaining anterior stabilizers if there is no competent subscapularis.

**Figure 6:**
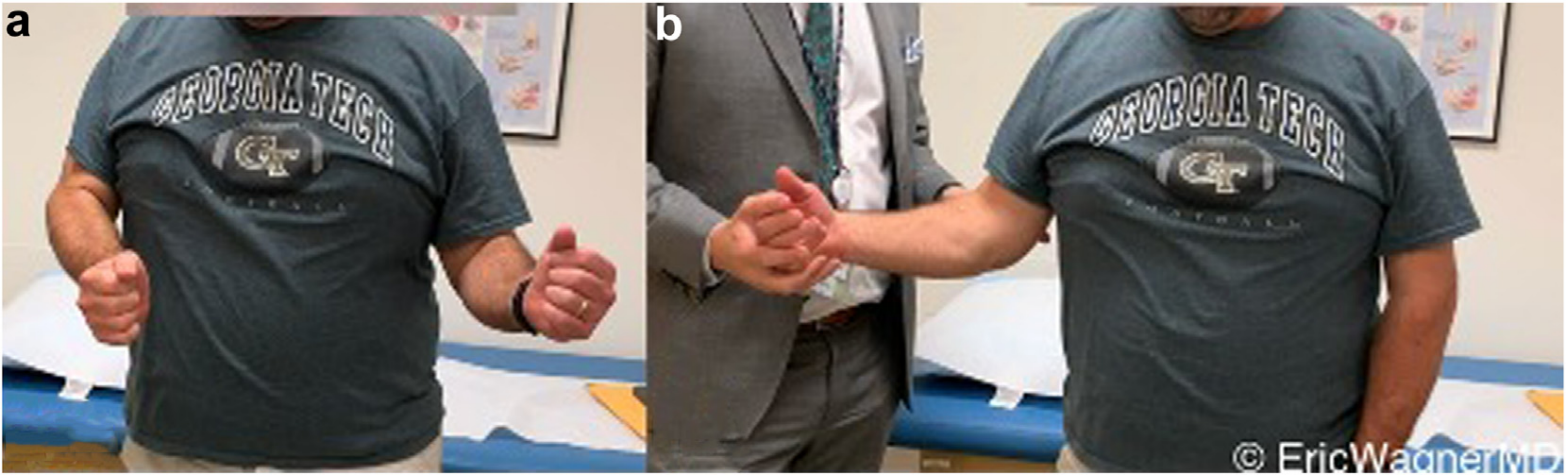
Patient with deficient posterosuperior rotator cuff and resultant external rotation lag. Note the difference in (**a**) active and (**b**) passive external rotation.

**Figure 7:**
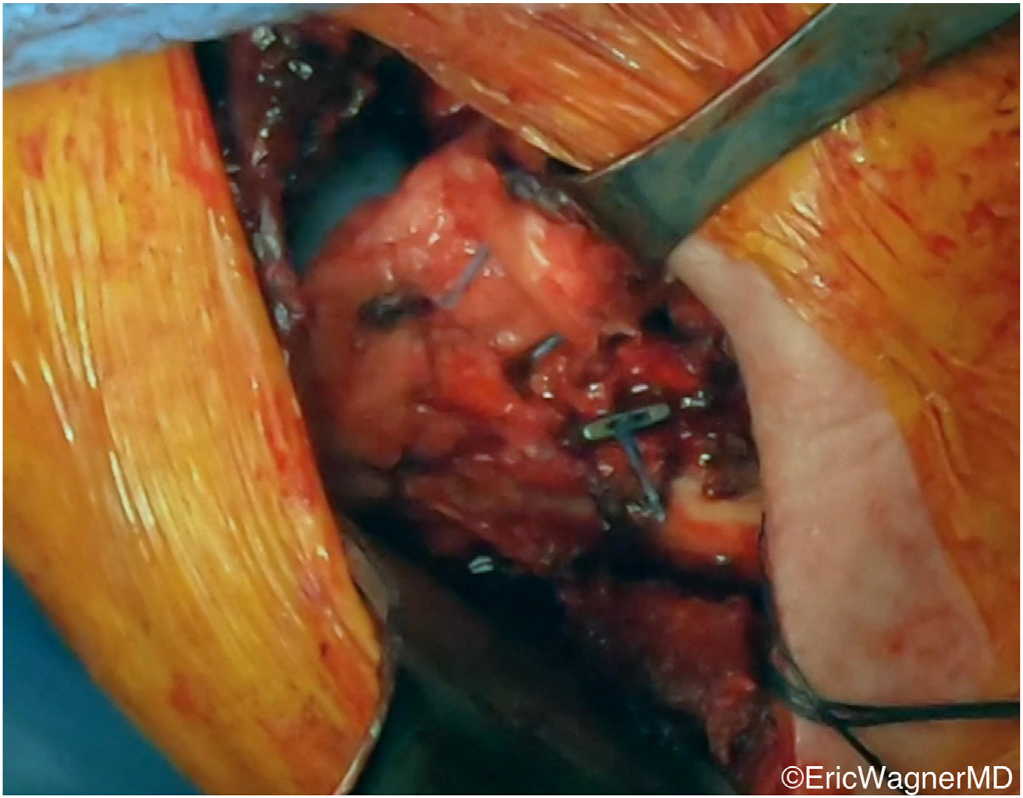
The tag sutures of the latissimus dorsi tendon have been passed from the posterior tuberosity to the anterior humerus through a bone tunnel, where they are secured by a dog bone button.

In the setting of a rTSA, we prefer the L’Episcopo technique if there is teres minor insufficiency and an ER lag sign preoperatively (Fig. 6). This can be performed through the deltopectoral interval, transfer around the humeral shaft just superior to the torn teres minor insertion, and anchored through a transosseous drill hole (Fig. 7). There is currently no consensus on whether the teres major needs to also be transferred or if the transfer of only the latissimus dorsi is sufficient. Kazum et al^25^ compared rTSA with isolated latissimus transfer to rTSA with combined latissimus and teres major transfer, finding no significant differences. Furthermore, several recent studies have questioned the need for these transfers entirely in the setting of the modern rTSA. Merolla et al^30^ found that the use of a lateralized humeral component provided equivalent restoration of ER as the L’Episcopo transfer. Young et al^55^ performed a randomized control trial comparing rTSA with and without the L’Episcopo transfer and showed similar improvements in function and PROMs. However, these studies were limited by their lack of power and diverse inclusion criteria. In the setting of true ER deficits and an incompetent teres minor, we believe the only way to restore true functional ER is with a L’Episcopo transfer.

### Lower trapezius transfer

The lower trapezius transfer (LTT) was first described by Elhassan et al^15^ in 2016 as an open procedure, and it was subsequently adapted in 2020 into an arthroscopically-assisted procedure.^13^ The LTT offers several key advantages over the L’Episcopo that have led to its recent increase in popularity. The LTT is a more anatomic transfer than the L’Episcopo, as the lower trapezius has a line of pull nearly identical to the infraspinatus. It may be technically easier to perform. In the setting of a deficient subscapularis, the LTT does not further compromise IR. The lower trapezius fire natively during ER and shoulder abduction,^44^ and thus does not require biofeedback training postoperatively, unlike the L’Episcopo.^28^ Finally, the LTT has been shown to have superior biomechanics to the L’Episcopo, with Muench et al^31^ finding lower compensatory deltoid forces and improved superior humeral head migration compared to the L’Episcopo. While not yet supported by strong long-term outcomes data like the L’Episcopo, short- and mid-term data on the LTT have been promising.^6^^,^^13^^,^^15^^,^^49^^,^^50^

**Figure 8:**
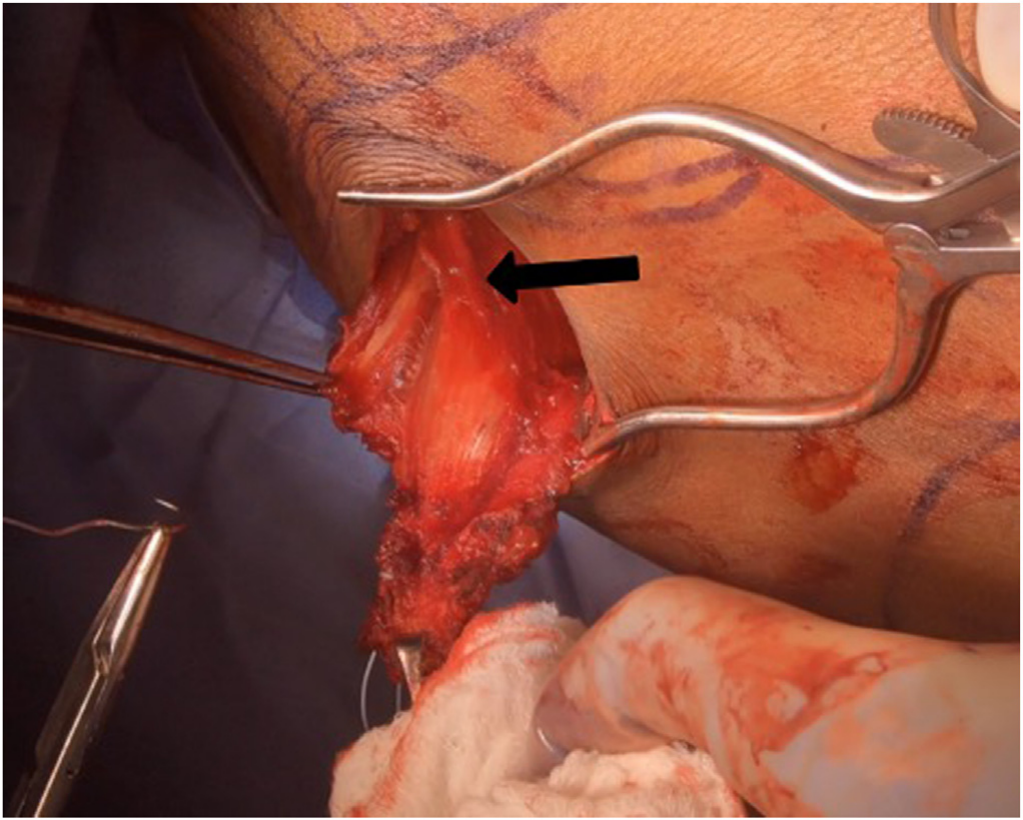
The lower trapezius has a robust tendon, which can be visualized on its deep surface. The black arrow identifies its pedicle, located 1.5 cm medial to the medial border of the clavicle, which must be protected.

The various surgical techniques are summarized by Clouette et al^6^ in their systematic review. Regardless of technique, the lower trapezius must first be mobilized from its insertion on the scapular spine, the tendon identified and tenotomized as distal as possible, and the lower trapezius muscle belly separated bluntly from the middle trapezius and underlying rhomboids (Fig. 8). Care must be taken to protect the pedicle, which inserts into the lower trapezius approximately 1.5cm medial to the medial border of the scapula. This pedicle can be inadvertently damaged during elevation and separation of the lower trapezius muscle belly.

**Figure 9:**
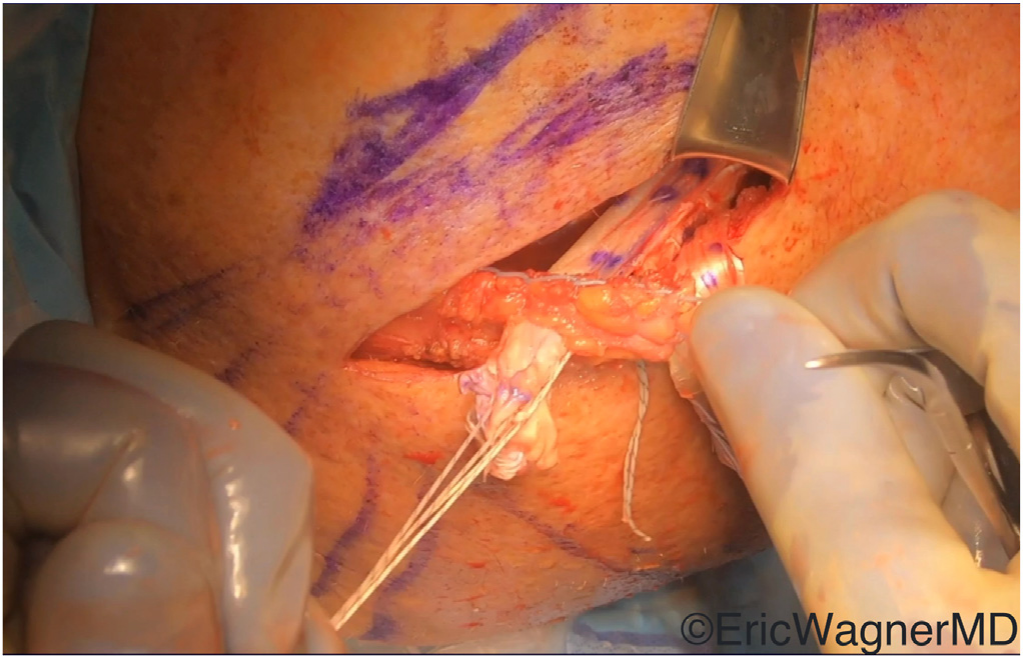
The Achilles tendon allograft has been arthroscopically anchored into the greater tuberosity. The proximal end is split, and half is joined to the lower trapezius tendon via the Pulvertaft weave. The other half (beneath the surgeon’s thumb) is overlayed and sutured to the lower trapezius tendon.

**Figure 10:**
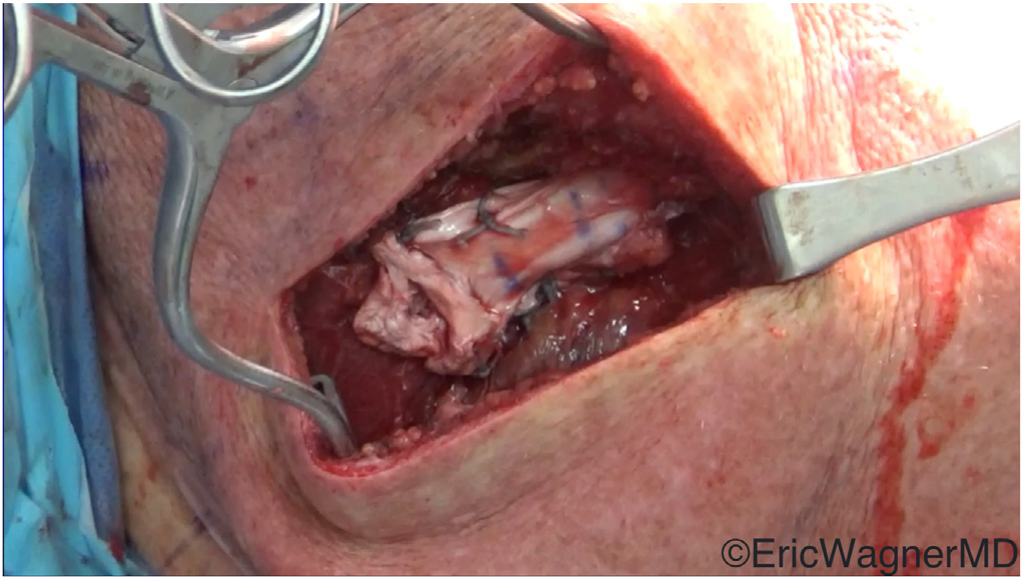
Final appearance of the lower trapezius transfer augmented with Achilles allograft.

If the infraspinatus tendon is intact, such as in a brachial plexus injury, the lower trapezius tendon can be tied directly into the infraspinatus tendon. More commonly, however, the infraspinatus tendon is not competent. In this case, the lower trapezius will need to be inserted onto the posterior or lateral greater tuberosity. The tendon of the lower trapezius does not have sufficient length to directly insert onto the tuberosity, so supplementation with a graft is required. Achilles allograft is frequently used to avoid donor site morbidity, but hamstring and peroneal tendon autograft have been described as well.^12^^,^^13^^,^^38^^,^^46^^,^^47^^,^^49^^,^^54^ The graft should first be anchored into the posterosuperior greater tuberosity, and the free end tunneled out of the posterior incision. Here, the lower trapezius tendon and graft can be joined using a Pulvertaft weave (Fig. 9). Tensioning should be performed with the arm in slight abduction and full ER. Fig. 10 demonstrates the final appearance of the transfer.

## Deltoid insufficiency

### Pedicled pectoralis major transfer

Shoulder arthroplasty, particularly rTSA, requires an intact deltoid for stability and functioning. Deltoid dysfunction has traditionally precluded both rTSA and aTSA. However, there are clinical scenarios where patients without adequate deltoid function have a prior arthroplasty in place or require arthroplasty for pain relief. For example, these include patients who have advanced cuff tear arthropathy in the setting of axillary nerve injury, deltoid atrophy, or brachial plexus injury, as well as patients who suffer an axillary nerve injury during an index arthroplasty. These scenarios can be challenging to manage and frustrating for patients due to the limited options available.

The pedicled upper pectoralis major transfer (UPMT) was first described in 1991 by Hou and Tai.^22^ It was subsequently utilized by several authors to restore shoulder abduction with good results.^9^^,^^29^ The technique was modified by Elhassan et al^16^ for use with reverse shoulder arthroplasty in the setting of deltoid dysfunction. Their only postoperative complications were two cases (2/31, 6.5%) of acromial fractures and resultant chronic pain. They found significant improvement in PROMs and ROM, particularly FF. This is consistent with a subsequent study by Hanneur et al,^27^ which found that the UPMT most effectively reconstructs the anterior deltoid. Burkhard et al^5^ similarly described its use after failed repair of an avulsed deltoid in a patient with an rTSA in place. They described significant improvements in functional outcomes and PROMs.

There are two variations of the surgical technique, depending on the indication for the transfer. One involves the clavicular and upper sternal heads in the setting of isolated axillary nerve palsies, while the other involves transferring the entire pectoralis major muscle in the setting of upper trunk brachial plexus injuries. The first surgical technique involves the separation of the clavicular and upper sternal heads of the pectoralis major, which will be transferred from the lower sternal head. The lower sternal head is preserved and remains functional due to its separate innervation from the middle and lower pectoral nerves. It is also technically easier, as the nerve to the lower sternal head runs through the pectoralis minor, limiting the transfer potential without also releasing the pectoralis minor.

**Figure 11:**
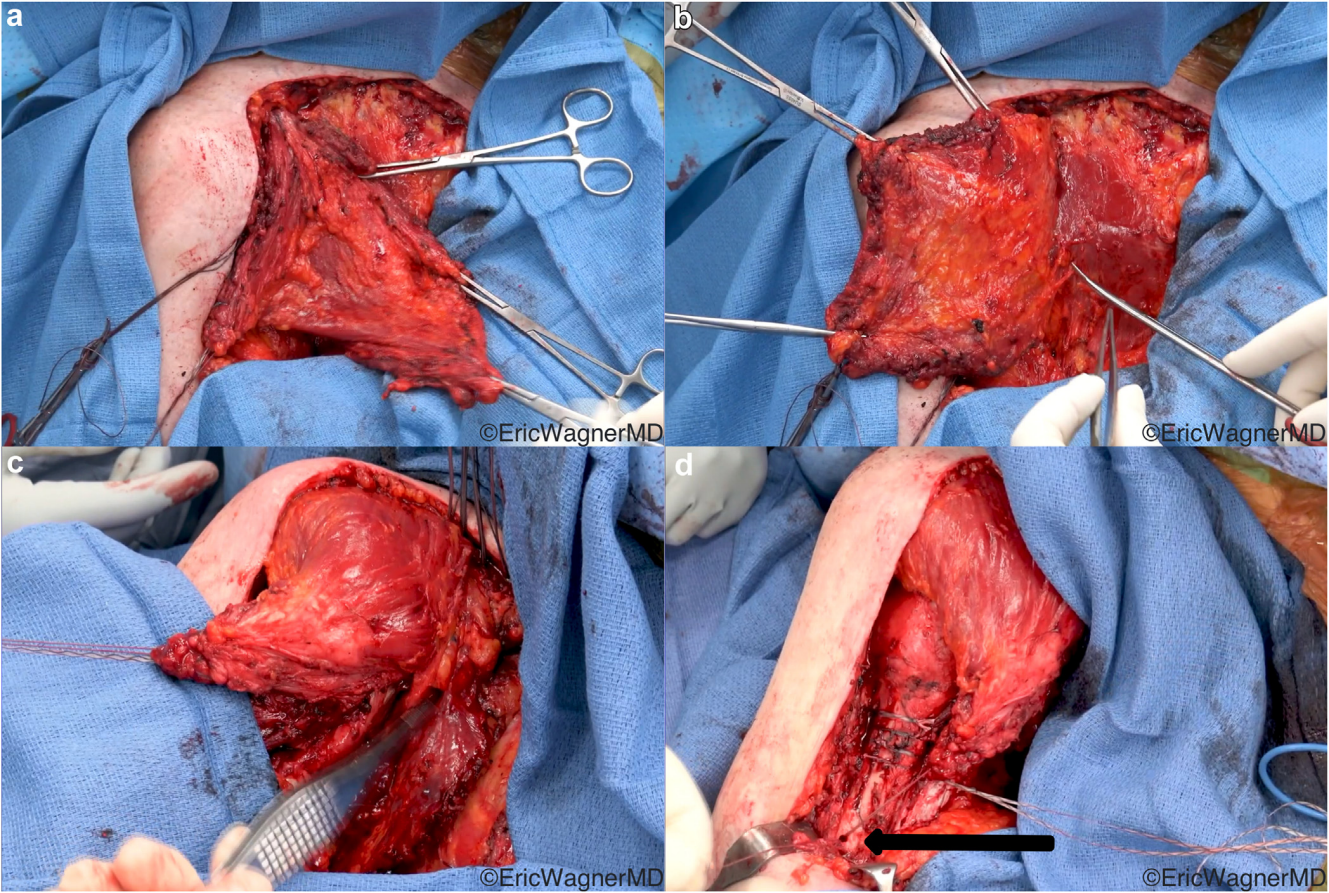
Intraoperative photos demonstrating the surgical technique of the pedicled upper pectoralis major transfer (UPMT) for deltoid insufficiency. (**a**) The clavicular head and upper sternal head are isolated and elevated off the anterior chest wall. (**b**) The flap is then flipped like the page of a book. The scissors point to the pedicle, which must be preserved. (**c**) It is then attached through bone tunnels into the lateral clavicle and acromion. (**d**) Finally, the humeral insertion is advanced distally to optimally tension the flap. The black arrow points to the bone tunnel to which the tendon will be advanced.

**Figure 12:**
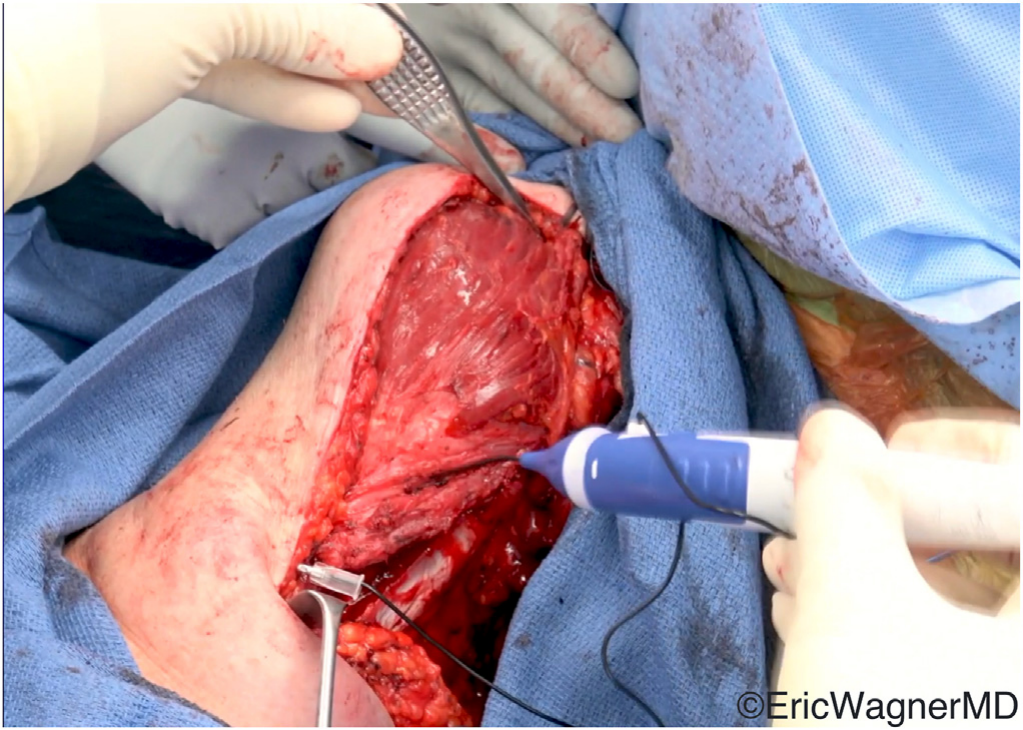
Final appearance of the pedicled upper pectoralis major transfer.

The clavicular and upper sternal heads are elevated off the clavicle, sternum, and anterior chest wall. The vascular pedicle enters the muscle inferior to the mid-clavicle, and care must be taken not to injure it when elevating in this area. At this point, the rTSA can proceed in the standard fashion. Once final implants are in, the flap is then flipped over a vertical axis, akin to turning the page of a book, allowing it to reach farther lateral without stretching the pedicle (Fig. 11). It is then attached proximally to prepared bone beds on the lateral clavicle and acromion. The humeral insertion can then be elevated and advanced to the lateral humeral shaft (Fig. 12).^5^^,^^16^

The UPMT can be combined with other transfers such as the LTT or L’Episcopo to restore ER if needed.^16^ When the clavicular head is insufficient in certain revision arthroplasty cases or upper trunk brachial plexus injuries, the entire pectoralis major is transferred. This requires elevating the pectoralis minor off the chest wall to de-tension the pedicle to the lower sternal head and enable the transfer of the lower sternal head.

While the early results of the pedicled PMT with rTSA are promising, there is a paucity of long-term outcome data. Additionally, the procedure can be technically challenging for surgeons unaccustomed to pedicled transfers around the shoulder. These may limit widespread adoption. However, the pedicled PMT currently represents one of only a few reconstructive options for patients with deltoid paralysis undergoing rTSA, so further investigation is warranted.

## Brachial plexus injuries

One unique indication of tendon transfers in the setting of upper trunk brachial plexus injuries that have not recovered involves a series of the above tendon transfers. In these cases, patients have deficient deltoid, supraspinatus, infraspinatus, teres minor, subscapularis, and clavicular heads of the pectoralis major muscles. However, the sternal heads of the pectoralis major and the trapezius are spared. Therefore, we prefer to perform a staged reconstruction for these patients.

The first stage involves reconstructing the deltoid insufficiency. This involves initially performing a pedicled pectoralis major transfer. Then, after 6 months of rehabbing and retraining this transfer, the second stage is performed, aimed at restoring shoulder stability and shoulder ER. This involves an rTSA with a medialized center of rotation and large glenosphere (to maximize the deltoid moment arm) and a LTT to the lateral tuberosity to maximize ER. This staged procedure allows for shoulder stability, restoration of functional ER and the ability to control their hand in space, and shoulder flexion to reach the top of the patient’s head.

## Proximal humerus bone loss

Massive proximal humerus bone loss can pose significant treatment challenges. In addition to bony augmentation, soft tissue reconstruction is critical for function and longevity. Proximal humerus megaprosthetic reconstruction can appropriately augment the deficient bone with an implant, but these have demonstrated poor soft tissue healing and a resultant high complication rate.^51^ Without coronal and axial force couples and rotational stability, these prostheses depend solely on the rTSA articulation for joint stability. This is often insufficient to maintain stability while also providing functional gains.

Allograft prosthetic composite (APC) rTSA has been described as one potential solution to this soft tissue problem.^7^^,^^23^^,^^41^^,^^42^ By incorporating the humeral stem into a proximal humerus allograft that still has tendon attachments, the surgeon is able to attach the patient’s intact tendons to the cadaver tendon insertions. Wang et al^51^ found better functional outcomes in this patient population with APC than with megaprosthesis. This was also seen in tumor reconstructions, where APC reconstructions had improved complication rates and functional outcomes.^23^ This is thought to be due to better soft tissue healing than can be achieved with a megaprosthesis.

Primary repair of the rotator cuff tendons is frequently not possible due to soft tissue destruction from multiple revisions or the patient’s primary pathology. The proximal humerus allograft has the rotator cuff tendons still intact, making the APC reconstruction well-suited to tendon transfers to restore shoulder function and stability. The surgical technique generally involves the placement of the APC rTSA implant, followed by the desired transfer(s).

These tendon transfers are technically similar to those described earlier in this article. The PMT or LDT can be used to reconstruct subscapularis function and anterior stability, and the L’Episcopo or LTT can be used to reconstruct the posterior rotator cuff. For the reasons discussed above, the authors prefer the LDT anteriorly and the LTT posteriorly, respectively. Notably, LTT in this setting differs from the more usual context of massive irreparable rotator cuff tears in that LTT with APC does not necessarily require Achilles tendon allograft augmentation since the proximal humerus has an intact infraspinatus tendon that can be tied into. The LTT does require a separate posterior incision, but it has the advantage over the L’Episcopo of being in phase for shoulder ER and having a more anatomic line of pull.^44^ Deltoid paralysis or insufficiency can also be reconstructed with a pedicled PMT, though no literature currently exists describing it in the setting of APC.

## Conclusion

Multiple tendon transfers about the shoulder have been described to reconstruct subscapularis deficiency, posterosuperior rotator cuff deficiency, deltoid deficiency, upper trunk brachial plexus injuries, and massive proximal humerus bone loss. These transfers can be performed in conjunction with reverse shoulder arthroplasty or, in some cases, anatomic shoulder arthroplasty, and they have generally been shown to improve functional outcomes in this setting. Shoulder surgeons should familiarize themselves with these techniques, as they can be utilized in challenging clinical scenarios where few other viable options exist.

## Disclaimers:

Funding: No funding was disclosed by the authors.

Conflicts of interest: Eric Wagner is a consultant for Stryker, Zimmer-Biomet, Osteoremedies, and Acumed. The other author, his immediate family, and any research foundation with which he is affiliated have not received any financial payments or other benefits from any commercial entity related to the subject of this article.

The editor making the decision to accept this paper for publication had no conflicts of interest related to the decision. Further, peer review of this paper was handled independently of any author of this paper.
